# Aging-Associated Changes in Hematopoiesis and Leukemogenesis: What's the Connection?

**DOI:** 10.18632/aging.100351

**Published:** 2011-07-02

**Authors:** Curtis J. Henry, Andriy Marusyk, James DeGregori

**Affiliations:** ^1^ Department of Biochemistry and Molecular Genetics, Integrated Department of Immunology, Department of Pediatrics, Program in Molecular Biology, University of Colorado Denver School of Medicine, Aurora, Colorado, USA; ^2^ Department of Medical Oncology, Dana Farber Cancer Institute; Department of Medicine, Harvard Medical School, Boston, MA, USA

## Abstract

Aging is associated with a marked increase in a number of diseases, including many types of cancer. Due to the complex and multi-factorial nature of both aging and cancer, accurate deciphering of causative links between aging and cancer remains a major challenge. It is generally accepted that initiation and progression of cancers are driven by a process of clonal evolution. In principle, this somatic evolution should follow the same Darwinian logic as evolutionary processes in populations in nature: diverse heritable types arising as a result of mutations are subjected to selection, resulting in expansion of the fittest clones. However, prevalent paradigms focus primarily on mutational aspects in linking aging and cancer. In this review, we will argue that age-related changes in selective pressures are likely to be equally important. We will focus on aging-related changes in the hematopoietic system, where age-associated alterations are relatively well studied, and discuss the impact of these changes on the development of leukemias and other malignancies.

## INTRODUCTION

The proportion of elderly people is progressively rising throughout the world, with the most pronounced increase in the developed world. Elderly people (>65 years old) are expected to comprise greater than 20% of the world's human population by 2050 [1]. This increase is posing major challenges for healthcare systems, as aging is associated with marked increases in a number of diseases, including most types of cancers [2-4]. With more than 80% of human cancers being diagnosed after the age of 50 [5], aging represents the single most important prognostic factor for many cancers, including lung, breast, colon, prostate, and certain leukemias [3, 6, 7].

Since the original proposal by Peter Nowell [8], the cancer research community has widely accepted the idea that initiation and progression of tumors are the results of clonal evolution, where increased genetic instability fuels the selection of clones with progressively increased fitness. Historically, the predominant focus in conceptualizing this clonal evolution has been on mutations, perhaps due to the implicit assumption that once a relevant oncogenic mutation occurs in a relevant cell type, clonal expansion will inevitably follow. However, unless somatic evolution of cancer cell populations represents a special case, it should follow the same Darwinian model as evolutionary processes in populations in Nature, whereby fitness is context-dependent rather than absolute and the “winners” in the evolutionary game are determined by the interplay between diversification of heritable types and environment-dependent selection forces (see **Box 1** for definitions). Indeed, a growing body of evidence suggests that the fitness effects of oncogenic alterations are highly context dependent [9-11] and that Darwinian evolution is a more accurate representation of somatic evolution of cancers than linear step-wise mutation-centric models [12, 13].

Box 1 **Definitions**

Organismal fitness: a measure of reproductive success (the ability of an organism to pass its genes on to future generations of that organism).
Cell fitness: a measure of the ability of stem/progenitor cells of a certain epigenotype/genotype to pass this type on to subsequent cell generations. For discussions here, we are concerned with the fitness of cells that maintain replicative potential. Cell fitness is in some ways a relative parameter, and dependent on the fitness of competing cells. Thus, the relative representation of a particular clone within a progenitor cell pool is proportional to its fitness. On the other hand, the fitness of stem and progenitor cells should also be comparable across individuals of different ages or genotypes, even if measurement of this relative fitness requires that these cells be placed in competition, such as following transplantation into a common host.
Adaptive: increases fitness (e.g. a mutation that increases cellular fitness would be adaptive).
Mutation: we will often refer to heritable epigenetic and genetic mutational changes generally as “mutations”.
Adaptive landscapes: the potential epigenetic and genetic changes that could alter the fitness of a cell population.

**Figure 1:**
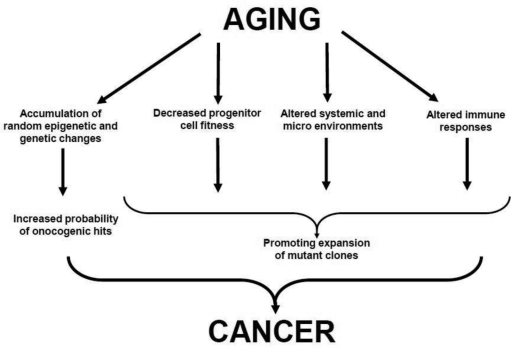
Proposed Links between Aging and Cancer See text for details.

Strong associations between aging and cancer are traditionally used to support the mutation-centric view of clonal evolution of cancers: aging leads to build up of random mutations, and since a) some of these random mutations are expected to activate cellular oncogenes or silence suppressor genes and b) transformation is thought to require cooperation between several oncogenic events, aging should translate into increased risk of cancer initiation [14]. On the other hand, aging is also associated with substantial changes both inside cells and in the cellular environment. In principle, these changes are likely to modify the ability of oncogenic mutations to drive clonal expansion. In fact, some of the age-related changes such as increased inflammation and decreased immune surveillance have been clearly implicated in carcinogenesis. In addition, intracellular changes such as telomere shortening as well as growth-inhibitory changes in the microenvironment could create a scenario for a strong selection for “oncogenic resistance to growth inhibitory conditions” [15]. We have previously proposed an Adaptive Oncogenesis Hypothesis (**Box 2**): populations of healthy young/progenitor cells possess high inherent fitness with little “room for improvement” [11, 16]. However, as cellular fitness drops due to cell-autonomous age-related changes or alterations in the microenvironment, certain mutations and epigenetic changes could alleviate the defects, leading to stronger selective expansion of the mutant clones. Therefore, the causal links between aging and cancer most likely includes both mutational components (diversification of heritable types through random mutations and heritable changes) and selection components (changes both inside and outside the cells that alter the adaptive landscape) (**Figure** **1**).

Box 2 **Adaptive Oncogenesis Hypothesis**

**Figure 2:**
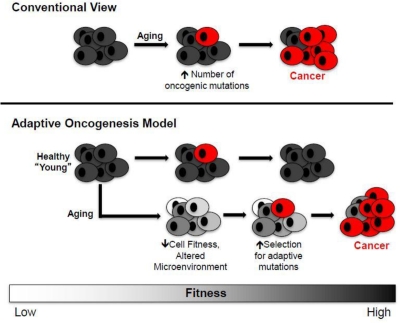
Conventional and Adaptive Oncogenesis Models for Tumorigenesis **Conventional View (top)**: Aging primarily contributes to increased cancers by facilitating the accumulation of oncogenic mutations (red cells), including activating mutations in oncogenes or genetic/epigenetic inactivation of tumor suppressor genes. **Adaptive Oncogenesis Model (bottom)**: The ability of an oncogene to induce cancer is context specific. In a healthy population, the ability of cells to effectively compete for niche space is high due to optimal progenitor cell fitness. Thus, this competition is inherently tumor suppressive. However, if cellular fitness decreases as a result of aging or environmental insults, the acquisition of an oncogenic mutation could be *adaptive* due to its ability to correct or circumvent defective cellular function. In this context, these cells would be selected for leading to carcinogenesis (oncogenically mutated and cancer cells are shown in red).

The most accepted explanation for the association between aging and cancer is that accumulating oncogenic mutations with age should facilitate cancer evolution [19]. Thus, according to this view, cancer development is limited primarily by the accumulation of oncogenic mutations (**Figure** **2, top**). But just as species evolution is driven by both mutation and selection, altered selection pressures with age should also contribute to oncogenesis. Thus, aged stem cell pools in an aged microenvironment should present a different “adaptive landscape” relative to young.

We have proposed that declining stem and progenitor cell fitness, whether due to aging or other insults, should increase selection for mutations (or epimutations) that are *adaptive* (i.e. that improve fitness) [10, 11, 16]. A change in the adaptive landscape should lead to selection for cells harboring advantageous traits (adaptive to the altered landscape), and these cells should be preferentially selected within their particular niche (**Figure** **2**). Aging or other insults move cells away from parameters that contribute to optimal fitness, thus creating opportunities for mutational adaptation. The acquisition of particular oncogenes in an aged background could be adaptive by either correcting or circumventing deficiencies in old cells. In addition, particular oncogenic mutations may render a cell resistant to aging-associated insults, such as chronic inflammation that promotes cellular senescence or apoptosis due to increased reactive oxygen species (ROS) production.

In contrast, a young healthy progenitor cell pool should be resistant to the fixation of trait-altering mutations. The evolution of long-lived multicellular organisms (like vertebrates) has necessitated the acquisition of potent tumor suppressive mechanisms, but this selection was limited to individuals who were likely to contribute to future generations [11]. Consequently, selection against cancer and other unpleasant manifestations of aging in the elderly has been quite weak during vertebrate evolution. As a simple example, mice in the wild rarely live past one year, and most age-associated tissue decline and increased cancer incidence occur well past this natural lifespan [20]. Thus, investment in tissue maintenance or tumor suppression beyond their natural lifespan would have required an investment of precious energy early in life, which could be better allocated towards survival and reproduction in youth. While George Bernard Shaw may have considered that “Youth is wasted on the young”, each species has ensured that youthful fitness is maximized for reproductive success. Stem cell fitness, which contributes to youthful robustness, will enhance reproductive success by limiting diseases of aging, including cancer.

The effects of aging are highly complex and span organismal, systemic and cellular levels [17]. Cancer development is similarly complex. Thus it would be naïve to expect a simple relationship between the two (**Figure** **1**). Numerous changes in aged individuals, including changes in cell type distributions in the bone marrow microenvironment, increased inflammation and decreased fitness of progenitor cell populations, should substantially impact on the expansion of oncogenically altered hematopoietic progenitor cell clones. Together with increased epigenetic/genetic diversity, these changes should increase the risk of cancers in old age. In this review we will focus on the hematopoietic system, as age-related changes in hematopoiesis are very well documented for both humans and mice [2, 18]. We will review age-related changes in the hematopoietic system and discuss how these changes might impinge on hematopoietic and non-hematopoietic malignancies.

### Aging Hematopoiesis

**Figure 3:**
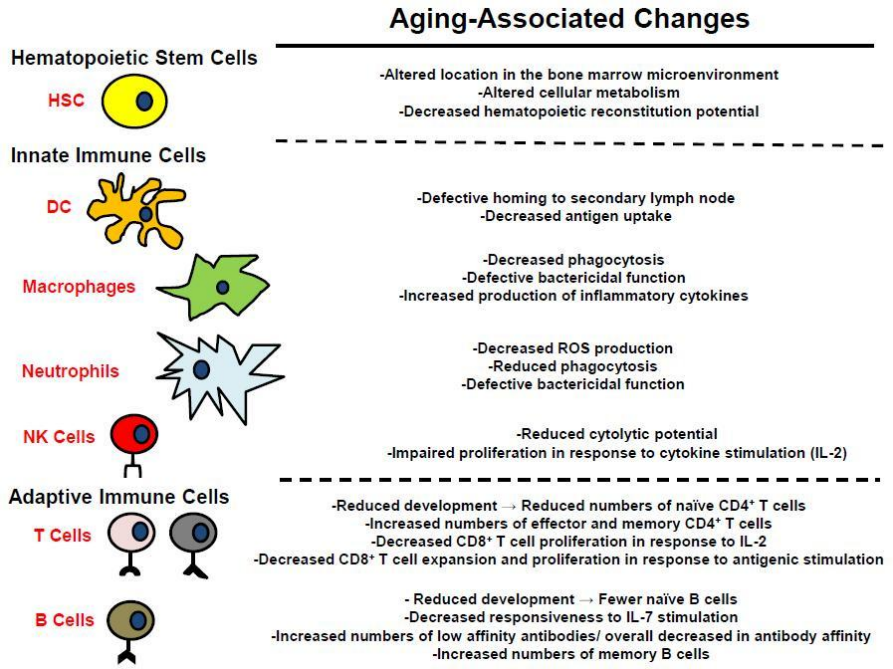
Alterations in the hematopoietic system associated with aging See text for details.

One of the more notable age-related changes is hematopoietic and specifically immunological decline [1, 21-25] (Figure 3). Maintaining the health of the growing elderly population is limited by the associated decline in immune function [23-25], impairing responses to pathogens and reducing vaccination efficacy. Decreased immune function is not compartmentalized; reduced immune cell function (and in some cases cell numbers) has been observed in both the myeloid and lymphoid lineages [23-25]. Furthermore, recent studies have indicated that these functional reductions result at least in part from aging-associated defects in hematopoietic stem cell (HSC) function, which are transferred to the their lineage-committed progeny [23, 25]. The causes of the aging-associated decline in HSC and hematopoietic cell function are still open for debate. Hematopoietic cell aging is associated with accumulating epigenetic and genetic changes, changes in the microenvironment of both immature and mature hematopoietic cells, and systemic changes such as inflammation. The relative contributions of these changes to altered HSC and mature hematopoietic cell function are still being explored. Moreover, a number of mechanisms have been proposed for how these changes might contribute to increases in age-associated cancers (Figure 1), and this review will focus on the multiple ways that aging could promote hematopoietic malignancies.

Hematopoiesis, which takes place in the bone marrow, is the production of all blood-derived cells from pluripotent HSC. Most of our current knowledge on the impact of aging on HSC function comes from studies using mouse models [23, 25-28]. These studies implicate both cell intrinsic and cell extrinsic factors towards impairmentsof age-dependent alterations in HSC behavior, although within animals both intrinsic and extrinsic influences are tightly interwoven and are sometimes difficult to disentangle [18]. Extrinsic factors, including changes in niche composition and hormone production, have been postulated to play a major role in declining HSC function with age [18, 29]. *In vivo*, multiphoton intravital microscopic analysis of HSC in the bone marrow of young and old mice has revealed that old HSC reside further away from the endosteum than young HSC progenitors, indicating age-related extrinsic changes in niche composition that could impact HSC function [30]. HSC normally reside in the HSC hypoxic endosteum microenvironment [30-32]. The effects of hypoxia on HSC function are just beginning to be delineated. It has been postulated that hypoxia regulates HSC self-renewal by promoting optimal turnover, which would limit potential accumulation of damage [32, 33], contributing to cell-intrinsic changes. Thus, residing further away from endosteum, by increasing oxidative damage to DNA, could increase the mutational load in HSC. In addition, it is believed that hypoxia limits the flow of extra-cellular fluids into the endosteum niche, which reduces HSC exposure to potentially hazardous toxins and pro-inflammatory cytokines, some of which are known to be tumor promoting [32-34]. Indeed, aging in mice is associated with increased detection of DNA strand breaks in HSC [35], and it is likely that increased mutational load contributes to increased initiation and progression of cancers in the elderly (whether mouse or human).

Moreover, another consequence of increasing mutational load in HSC (as in other stem cell types) should be the reduction in stem cell fitness, as DNA damage is more likely to negatively impact on cellular functions than improve them. Indeed, aging-related, cell autonomous changes have also been shown to contribute to altered HSC function [28, 36-38]. When highly purified long-term HSC (LT-HSC) from young or old mice are used to reconstitute young irradiated recipient mice, old LT-HSC are 2 to 4-fold less efficient at reconstitution per-HSC, and their production is skewed in favor of myelopoiesis, with accompanying reductions in lymphopoiesis (to be dealt with below) [27, 36, 38-40]. Thus, as defined above, old HSC are less “fit”, in that young competitors are better able to contribute to hematopoiesis. Aged mouse HSC also exhibit decreased homing and engraftment [39, 41]. And as described above, altered stem cell fitness should increase selection for adaptive oncogenic mutations (**Figure** **2**), increasing the chances that stem cells bearing particular oncogenic mutations expand within competitive HSC pools in an aged bone marrow microenvironment. Therefore, aging-associated alterations in HSC localization and mutational load should not only increase the *frequency* of oncogenic mutations, but *selection* for particular oncogenic mutations.

Finally, the hematopoietic system may attempt to compensate for decreased HSC function (and decreased production rates for mature progeny) by increasing the size of the HSC compartment, as indicated by the observation that the number of phenotypic HSC in C57Bl/6 mice increases with age [36, 38, 39, 42-44]. Such increased homeostatic mechanisms, both through increased cytokine levels and perhaps increased turnover rates for HSC and other progenitors, should contribute to higher chances for mutation fixation. Larger numbers of HSC should also increase the target size for oncogenic mutations. However, increased HSC numbers have not been observed in all mouse strains; BALB/c and DBA/2 do not show an age-dependent increase in the numbers of phenotypic HSC [45-47], but may still experience other homeostatic mechanisms. Although the effects of aging on human HSC have not been as extensively studied, human HSC function also appears to be affected by aging given the observation that the proliferative potential of human HSC declines with age [48]. In addition, bone marrow from older humans is less efficient at reconstituting recipients when compared to the reconstitution capacity of bone marrow derived from younger patients [49]. In summary, based on experiments in mice and more limited observations in humans, it is clear that a number of age-associated alterations in the HSC compartment, due both to extrinsic and intrinsic factors, could contribute to increased frequencies of hematopoietic malignancies in the elderly.

### Aging Innate Immunity

In aged mice, it has been demonstrated that a developmental shift occurs, with reduced lymphopoiesis in favor of greater myelopoiesis. Microarray analyses of HSC isolated from old mice reveals a gene expression profile consistent with this age-dependent bias towards production of myeloid cells [36, 38]. The myeloid bias appears to be at least in part cell-autonomous, as adoptive transfer of young and old HSC into young, irradiated recipients recapitulates the age-dependent bias towards myelopoiesis [1, 36, 50]. This bias appears to be at least in part mediated by a shift towards greater frequency of myeloid-biased relative to lymphoid-biased HSC [42, 51, 52], and these differentially biased HSC can now be prospectively isolated and shown to transfer their developmental bias to recipient mice [37, 53, 54]. It is important to note that while myelopoiesis becomes favored overlymphopoiesis in old mice, myeloid progenitors are not “more fit” relative to young. Myeloid and B-cell progenitors appear to compete for common niches [55], and mouse and human myeloid progenitors exhibit age-dependent reductions in progenitor activity [56, 57], while mouse B-progenitors show even greater reductions in function (thus, providing myelopoiesis with a relative advantage). Moreover, elderly humans exhibit decreased developmental potential for both lymphoid and myeloid progenitors [26, 57].

Decreased cellular function in old age has been documented in dendritic cells (DC), neutrophils, and macrophages (**Figure** **3**) [23, 58]. DC are the professional antigen presenting cells of the immune system, and are the principle cells responsible for priming naïve CD4^+^ and CD8^+^ T cells [23]. The potency of DC as primers of naïve T cells declines with age [23]. This decrease has been shown to result from reduced migration of DC to secondary lymphoid organs following stimulation and defective antigen uptake, which results in decreased presentation to naïve T cells.

Natural killer (NK) cells, which protect against tumors and virally infected cells, are also impacted by the aging process in humans [23]. While their numbers actually increase with age (despite a reduced proliferative response to IL-2), their cytolytic potential decreases in aged populations. Aging-associated reductions in DC and NK function may contribute to increasing cancer incidence with age due to decreased activation of cancer-specific T cells by DC and impaired NK mediated tumoricidal activity.

In addition to the decreased innate cellular function that accompanies aging, older humans typically present a subclinical inflammatory status termed “inflamm-aging”, which is characterized by increased tumor necrosis factor-alpha (TNF-α), interleukin-6 (IL-6), and interleukin-1β (IL-1β) in the plasma [23, 59]. Inflamm-aging may also contribute to alterations in HSC with age [38, 53]. The increase in the circulating levels of inflammatory cytokines reflects cell autonomous changes in myeloid gene expression resulting in microenvironmental changes that could favor tumor initiation and progression [23]. Indeed, chronic inflammation is a well defined contributor to tumorigenesis, with tumor associated macrophages (TAMs) playing a key role in driving tumorigenesis [59]. The cytokines produced by these cells, such as TNF-α, could influence cancer progression both by promoting proliferation of oncogenically initiated or more advanced tumor cells and by inducing death of non-malignant cells (thus stimulating compensatory proliferation) [34, 60]. Inflammation may also create a hazardous microenvironment, with increased ROS and cell death, thus promoting selection for oncogenic events that confer resistance or are otherwise adaptive to this altered microenvironment. Thus, aging-associatedalterations in the innate immune system not only increase susceptibility to pathogens, but may also be critical in the evolution of many cancers, which can be promoted by inflammatory mediators.

### Aging Adaptive Immunity

Age-associated impairment of the adaptive immune system, which is composed of T cells and B cells, is observed in both mice and humans (**Figure** **3**) [1, 27], and could impact cancer development in several respects. In mice, the production of naïve CD4^+^ T cells (T helper cells) declines with age [61]. CD8^+^ T cell function also declines with age [23]. Specifically, naïve CD8^+^ T cells isolated from aged individuals proliferate less when stimulated with IL-2, and their expansion and differentiation into effector T cells upon antigenic stimulation is reduced when compared to naïve CD8^+^ T cells isolated from young individuals. Although cell-autonomous changes in HSC also contribute to age-associated reductions in T-lymphopoiesis [62], reductions in the numbers of T cells are mainly attributed to thymic involution that occurs with aging [1, 61]. Aging-associated changes in the thymus lead to a decline in functional cortex and medulla, which is replaced by fat [21, 61]. Decreased T cell maturation in the thymus may actually contribute to *reduced* T-acute lymphoblastic leukemia (T-ALL) incidence in the elderly (T-ALL is primarily a childhood cancer), as substantially reduced T cell precursor numbers would reduce the target population for acquisition of oncogenic mutations.

Aging-associated alterations in T lymphocyte numbers and function lead to increased susceptibility to pathogenic infections and a decreased ability to effectively vaccinate the elderly; however, the importance of T lymphocytes in the control of aging-associated cancers is somewhat unclear. Increased cancer incidence has been observed in immunodeficient mice, as well as humans that are immunocompromised, such as for Karposi's sarcoma, non-Hodgkin's lymphoma, and cervical cancer in AIDS patients [63-66]. Declining adaptive immunity could increase the incidence of these particular cancers because they are virally associated; however, for non-virally associated cancers, the role of the adaptive immune system in controlling cancer initiation is still controversial. It has been shown that immunodeficient mice develop more carcinogen-induced and spontaneous cancers than wild-type mice [67, 68]. However, straightforward interpretation of studies on mice with compromised adaptive immunity is complicated by the complex nature of pro- and anti-tumorigenic interactions with the primary and adaptive immune systems [69]. Nonetheless, mouse models have revealed that the adaptive immune system is important for the initial elimination of cancer cells, and that T cells play a role in equilibrium (the phase where oncogenic cells are not detectable) and escape phases (relapse) of certain forms of cancers like sarcomas [67]. However, in an inducible melanoma model, it was found that tumor-initiated inflammation could suppress the adaptive immune system's ability to delay the development of aggressive melanomas [70]. Therefore, mouse models indicate that the adaptive immune system indeed plays a role in the control of certain forms of cancer; however, inflammation can suppress the protective roles of the adaptive immune system. Since aging is associated with an inherent increase in inflammation and declining adaptive immune function, these changes could cooperate to reduce adaptive immunity, including against malignancies, in old age.

For B cell maturation, cell intrinsic and extrinsic changes both contribute to reductions in production and function of B cells [27, 71, 72], which could in part underlie increased disease susceptibility, reduced responses to vaccination, and increased cancer incidence in aged populations. Prior to their peripheral migration to secondary lymphoid organs, B cells develop in the bone marrow, differentiating through multiple stages [27]. Studies conducted in mice have identified decreased frequencies of common lymphoid progenitors (CLP)/ early B cell progenitors (EBP), pre-pro B cells, pro-B cells, and pre-B cells in the bone marrow of old mice [71, 73-75], which are due in good measure to cell-intrinsic changes in HSC [36, 50]. *In vitro* studies show that aged mouse pro-B-cells exposed to limiting interleukin-7 (IL-7) proliferate less than young pro-B-cells, despite similar IL-7-receptor (IL-7R) expression levels [76, 77]. Moreover, administration of antagonistic anti-IL-7 antibody to young mice has been shown to recapitulate the peripheral B-cell repertoire skewing observed in old mice [78]. Thus, the decrease in B-lymphopoiesis in aged mice may partially be attributed to a decrease in the ability of B-progenitors to respond to IL-7 stimulation.

In addition to the decrease in the total number of B cells found in the periphery of aged mice, their function also declines in an age-dependent manner (**Figure** **3**) [23, 27, 71]. Specifically, aged mouse B cells exhibit increased production of low affinity antibodies due to decreased isotype switching. In humans, the aging-associated decline in B cell lymphopoiesis appears less drastic compared to mice, and it has been demonstrated that B cell lymphopoiesis in humans is less dependent on IL-7 stimulation [79, 80]. The decline in humoral responses in elderly people is attributed to changes in the composition of the cells comprising the B cell repertoire [23, 81, 82]. Fewer naïve B cells are found in elderly populations compared to the numbers found in young adults, and these cells are replaced in the B cell pool by antigen experienced memory cells. Also, there is a decrease in overall antibody affinity found in elderly populations due to a general isotype switching from IgG to IgM antibodies [23, 83, 84]. Although the effects of aging on B-lymphopoiesis differ between mice and humans, there is significant overlap in the decline in B cell function found in aged population from both species. While reductions in B-lymphopoiesis in old age could contribute to reduced tumor immunesurveillance, as will be discussed below, there has been more debate about the implications of perturbed B-cell development towards leukemogenesis.

### Emerging Models for Aging and Cancer

**Figure 4:**
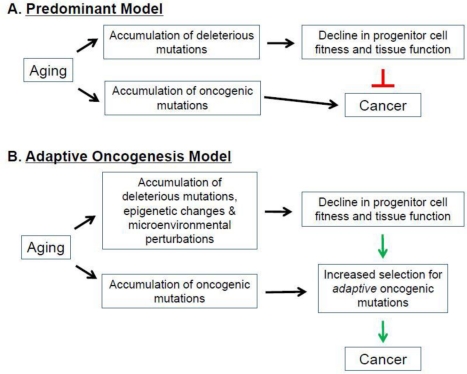
Aging, Cancer, and Selection of the Fittest. (A) Predominant Model. (B) Adaptive Oncogenesis Model See text for details.

The strong association between aging and cancer has been a focus of intensive research, with the current paradigm suggesting that the accumulation of oncogenic mutations is the rate-limiting step in the initiation and progression of cancer [14]. It is also frequently argued that aging is in part the consequence of organisms attempting to repress cancer evolution [1, 14, 26, 85-90]. In this view, aging and tissue decline limit cancer development, through telomere shortening and tumor suppressor gene action. Thus, preventing cancer during youth (when animals are most likely to contribute to future generations) requires mechanisms, such as limited telomere maintenance, which contribute to aging phenotypes later in life. Thus, aging is thought to in part represent a cost of tumor suppression (**Figure** **4A**).

According to this model, aging-associated events (ie., telomere shortening) increase the activation of tumor suppressor genes, which inhibits cancer at the expense of accelerating the aging process [1, 26, 86, 87, 91, 92]. Indeed, aging-associated reductions in telomere length and function activate p53, a critical tumor suppressor [26, 93]. Chronic activation of p53 results in a suppression of DNA replication, decreased cellular proliferation, and ultimately cellular senescence or death [89, 94, 95] (however, note that increased p53-dependent tumor suppression can be obtained without accelerated aging [96, 97]). In addition to p53, expression of the INK4A locus-encoded p16and Arf tumor suppressor genes also increases with age in certain hematopoietic compartments [98]. Moreover, increases in p16 and Arf have been shown to contribute to the aging-associated decline in lymphopoiesis [98]. Decreasing the expression of these tumor suppressors in aged lymphoid progenitors reverses the senescent phenotype, resulting in an increased susceptibility to transformation [98]. These models provide evidence that increased expression of tumor suppressor genes is associated with aging in certain cellular populations. But if tumor suppressive mechanisms are elevated in aging tissues, then why is there such a strong association between age and the increased incidence of most cancers, and why do interventions that delay aging (like caloric restriction) also delay cancer occurrence [99]?

The hematopoietic system has been a valuable organ for studying the relationship between aging and cancer [2]. In humans (as for other vertebrates), most cancers, including some leukemias, show age-dependent increases in incidence [2, 7]. The most common form of chronic leukemia in elderly people is B-cell chronic lymphoblastic leukemia (B-CLL) [100], and the most frequent acute leukemia is acute myelogenous leukemia (AML) [101]. In addition, leukemias induced by the oncogene Bcr-Abl (Ph^+^), including chronic myelogenous leukemia (CML) and Philadelphia Chromosome^+^ B-cell acute lymphoblastic leukemia (Ph^+^ B-ALL), show age-dependent increases in incidence [102]. This age-dependence is more striking for CML, with the majority of diagnosis occurring in individuals over 50 years old [103]. For Ph^+^ B-ALLs, the average age of diagnosis is 50 [104, 105], and the increased incidence with advancing age is less dramatic than for CMLs.

The correlation of aging with leukemias has been studied extensively in mouse models [56]. In general, these studies argue that the increase in aging-associated myeloid leukemias in mice results from aging-associated impairment of lymphopoiesis and increases in myelopoiesis [37, 56, 71, 106]. According to these models, the decline in the number and function of lymphoid progenitors reduces the probability of a B lymphoid progenitor acquiring an oncogenic mutation [2, 56, 71, 98]. The increase in myelopoiesis would in turn accelerate the possibility of acquiring a transforming event in a myeloid progenitor leading to the observed aging-associated increase in myeloid lineage leukemias. Also, these models predict that increased expression of tumor suppressor genes in aged lymphoid progenitors prevents transforming events from frequently occurring in this lineage.

As myeloid leukemias are common in elderly humans, these investigators have argued that these results are consistent with leukemia development in the lineage (myeloid) that remains dominant (i.e. more fit) in mice [2, 5, 56, 71, 98, 107]. But if the dominant lineage is the most leukemogenic, one should expect mice to develop myeloid leukemias. But instead mice mostly develop lymphoid malignancies in old age [108, 109], coinciding with the reduced fitness of lymphoid progenitors. Unlike mice, aging humans exhibit pan-hematopoietic decline, including in the myeloid lineage [26, 57]. This correlates with an increase in both myeloid (e.g. AML) and lymphoid leukemias (e.g. B-CLL and Ph^+^ ALL) which are common in the elderly. Indeed, a major risk factor for AML are myelodysplastic syndromes and other bone marrow failure syndromes (with reduced HSC and myeloid progenitor fitness), which have been proposed to increase selection for adaptive oncogenic mutations, thus increasing the frequency of AML development [110]. Thus, *reduced* fitness in particular progenitor cells actually coincides with increased malignancy development with age, and may at least in part explain species-specific differences in the types of leukemias that develop.

As introduced above, we have proposed an alternative model, dubbed Adaptive Oncogenesis, which proposes that youth and high tissue fitness are actually tumor suppressive [10, 11, 16] (**Figures** **2** **and** **4B**). This model postulates that long-lived multicellular organisms have evolved stem cell populations with high fitness, not only as a means of efficiently maintaining a tissue, but also because high fitness in a cell population will oppose somatic cell evolution. As in animal populations well adapted to their environments, stabilizing selection should limit changes that improve fitness in a population of stem cells with high fitness. Competition in a young, healthy stem cell pool should serve to maintain the status quo, preventing somatic cell evolution. But in stem cell pools impaired by aging or other insults, the adaptive landscape will be dramatically altered [10, 11]. The fitness of the stem cell pool will be reduced, allowing selection for mutations and epigenetic events that improve fitness of somatic cells. We postulate that, from an evolutionary biological perspective, aging is associated with increased cancer incidence because there is minimal selection against cancer at ages where an animal is unlikely to contribute to future generations.

We recently obtained support for this model in the context of Bcr-Abl-initiated leukemias in aged hematopoietic systems [40]. Consistent with previous studies, we showed that aging results in decreased hematopoietic progenitor cell fitness, exhibited by a reduction in HSC frequency and decreased B-lymphopoiesis. Pro- and pre- B cell progenitors isolated from aged mice exhibited defective activation of Akt and STAT5, which lie downstream of the IL-7R. In contrast, the phosphorylation of STAT3 and Erk was equivalent to levels observed in B cell progenitors isolated from young mice, indicating that there is not a general decrease in all signaling pathways in old B-progenitors. The expression of Bcr-Abl in old mice, and in mice under IL-7-neutralized conditions, restored kinase signaling to Akt and STAT5. This leads to an increased selection of Bcr-Abl expressing cells within B-progenitor pools, and ultimately increased leukemogenesis *in vivo* [40]. Notably, the expansion of Bcr-Abl-expressing cells within aged B-progenitor pools can be countered by transplantation of young competitors, highlighting the importance of hematopoietic progenitor pool fitness in preventing leukemia. Moreover, the early expansion of Bcr-Abl-expressing progenitors in aged lymphoid progenitor populations is polyclonal, and thus pre-accumulated oncogenic mutations in old progenitors are unlikely to account for age-dependent selection for Bcr-Abl expression (but note that a different study showed that the age of the target cell for Bcr-Abl induced B-ALLs does contribute to age-dependence [111]). Instead, since aging results in reduced receptor-mediated kinase signaling in B-progenitors, and Bcr-Abl expression can restore this signaling, we would argue that an important contribution of aging towards Bcr-Abl-initiated leukemogenesis is the increased selection for Bcr-Abl in signaling-deficient B-progenitor pools [40].

Thus, we propose that in young lymphoid progenitors, efficient receptor signaling (such as via IL-7R) provides the “right amount” of activation of downstream effectors for optimal B-progenitor fitness. In this context, the activation of downstream effectors by Bcr-Abl would lead to excessive signaling, beyond the levels needed for optimal fitness. But in old B-progenitors, receptor signaling is deficient, and thus Bcr-Abl would be adaptive, in part by increasing signaling via effectors shared by receptors like the IL-7R. To put it simply, the reduced fitness of aged progenitor cell pools creates “room for improvement”.

## CONCLUSION

The increasing proportion of elderly people in populations worldwide and associated increases in rates of cancers pose substantial challenges to biomedical research and medical care, making the task of deciphering mechanisms linking the two a top priority. Since cancer is generally considered a disease of the elderly, a more comprehensive understanding of how aging alters cellular function, and how alterations in cells and tissues impact on carcinogenesis, should contribute to the development of more efficacious therapeutic and prevention strategies for cancer [112]. Studies in mice and humans have revealed that aging is characterized by drastic reductions in immune cell function, which subsequently leads to decreased immune surveillance. This immunocompromised state has mainly been attributed to alterations in HSC and hematopoietic development (and in some cases cell numbers), and aging-associated thymic involution. Deficiencies in these compartments result in reduced myeloid and lymphocyte cellular functions and decreased T and B cell diversity in the periphery. Importantly, aging is also associated with increased incidence of many cancers, including hematopoietic malignancies, and in this review we have discussed the many possible connections between aging and cancers.

The most widely accepted explanation for the association between aging and cancer is that accumulating oncogenic mutations (and epimutations) with age should facilitate cancer evolution. Thus, according to this view, cancer development is limited primarily by the accumulation of oncogenic mutations. But just as species evolution is driven by both mutation and selection, altered selection pressures with age should also contribute to oncogenesis. Thus, the aged microenvironment should present a different adaptive landscape relative to a young microenvironment. Mutation and selection centric explanations are not mutually exclusive; instead they can be expected to act in parallel to contribute to the increase rates of cancers with old age. Both aging and cancer are highly complex and their causes are certainly multifactorial, and models to explain their clear association will also need to be multifaceted. We have reviewed the various changes within the hematopoietic system with aging that are expected to contribute to increased cancer incidence in the elderly, including accumulating mutations and epigenetic changes, increased inflammation, decreased adaptive immunity, altered numbers and fitness of progenitors, and other alterations that could change the adaptive landscape.

Exciting recent finding suggest that at least some of the effects of aging are not irreversible, as previously thought, and it is possible to rejuvenate cells and tissues [92, 113]. Beyond the obvious potential health benefits of the reversal of age-dependent tissue regeneration, the ability to rejuvenate tissues should also allow researchers to disentangle mutational and selection components in age-related increases in cancers. If the selection component plays a major role in the age-related spike in cancers, one should expect decreases in cancer rates upon reversal of tissue degeneration. A clearer understanding for how aging-associated changes within the hematopoietic system influence cancer development will be essential for the development of preventative and therapeutic strategies.
